# Association of Cytochrome *CYP1A1* Gene Polymorphisms and Tobacco Smoking With the Risk of Breast Cancer in Women From Iraq

**DOI:** 10.3389/fpubh.2018.00096

**Published:** 2018-04-11

**Authors:** Hassan M. Naif, Mohammed A. I. Al-Obaide, Hayfa H. Hassani, Abdualghani S. Hamdan, Zainab S. Kalaf

**Affiliations:** ^1^Molecular and Medical Biotechnology, College of Biotechnology, Al-Nahrain University, Baghdad, Iraq; ^2^School of Medicine, Texas Tech University Health Sciences Center, Amarillo, TX, United States; ^3^Biology Department, University of Baghdad, Baghdad, Iraq; ^4^Al-Russafa Health Directorate, Ministry of Health, Baghdad, Iraq

**Keywords:** *CYP1A1* genotype, breast cancer, smoking, genetic polymorphism, Iraq

## Abstract

**Background:**

*CYP1A1* gene polymorphisms and tobacco smoking are among several risk factors for various types of cancers, but their influence on breast cancer remains controversial. We analyzed the possible association of *CYP1A1* gene polymorphisms and tobacco smoking-related breast cancer in women from Iraq.

**Materials and methods:**

In this case–control study, gene polymorphism of *CYP1A1* gene (*CYP1A1m1*, T6235C and *CYP1A1m2*, A4889G) of 199 histologically verified breast cancer patients’ and 160 cancer-free control women’s specimens were performed by using PCR-based restriction fragment length polymorphism.

**Results:**

Three genotype frequencies (TT, TC, and CC) of *CYP1A1m1T/C* appeared in 16.1, 29.6, and 54.3% of women with breast cancer, respectively, compared with 41.2, 40, and 18.8% in the control group, respectively. *CYP1A1m1* CC genotype and C allele were significantly associated with increased risks for breast cancer in patients (54.3 and 69%, respectively) compared with controls (18.8 and 39%, respectively). While the three genotype frequencies (AA, AG, and GG) of *CYP1A1m2A/G* were detected in 20.1, 31.2, and 48.7% in patients compared with 46.3, 40.6, and 13.1% in controls, respectively. The frequency of GG genotypes and G allele was significantly higher in patients (48.7 and 64%, respectively) than in the controls (13.1 and 33%, respectively). Smoking women having either CC or GG genotypes showed a highly significant association with increased risk of breast cancer [odds ratio (OR) = 1.607, 95% confidence interval (CI) 0.91–1.64, *p* = 0.0001, and OR, 1.841, 95% CI, 0.88–1.67, *p* = 0.0001, respectively]. On the other hand, the T and A alleles of predominantly seen in healthy smoking women (83 and 85%, *p* = 0.0001, respectively).

**Conclusion:**

These findings indicated that both C and G alleles of *CYP1A1m1* and *m2* were significantly associated with elevated risk of breast cancer in Iraqi women, while the T and A alleles were predominantly seen in healthy controls which may indicate their protective role. The C and G association with breast cancer incidence was more prevalent among tobacco smoking patients. These polymorphisms may be used as biomarkers of breast cancer in women from Iraq.

## Introduction

It is known that a combination of genetic and environmental risk factors such as hypertension, tobacco use, obesity, and exposure to estrogen and polycyclic aromatic hydrocarbons were shown to be involved in breast cancer (1, 2). There are multiple risk factors involved in mediating breast cancer such as DNA damage, cell proliferation, and mutation of the estrogen receptor gene as well as reproductive issues (3–6).

Cytochrome P450 (*CYP*) is a superfamily of enzymes, including *CYP1A1*, which catalyze several reactions such as drugs, cholesterol, steroid including estrogen and environmental pollutant breakdown and metabolizes several procarcinogens into active human carcinogens (4, 5, 7). The *CYP1A1* gene is thought to be involved in breast cancer because of its role in the metabolism of the polycyclic aromatic hydrocarbons and in the oxidative metabolism of estrogens that might increase the risk of oxidative stress and cancer (8–10). Polymorphisms of the *CYP1A1* gene have been associated with increased aryl hydrocarbon hydroxylase activity which in turn may change individual’s susceptibility to breast cancer by either increasing risk in women in different parts of the world (10–14), or had no effect on the disease outcome (15–17).

Tobacco smoking is among the main preventable risk factors for several different diseases. The correlation between active tobacco smoking and breast cancer showed an increased risk among women with certain *CYP1A1* mutant genotypes (18, 19). The interaction between several compounds in tobacco smoke such as polycyclic hydrocarbons and *CYP1A1* genotypes has been reported to influence risk of breast cancer (20–24). However, such association needs further investigation of women with breast cancer in Iraq, because of inconclusive reports (25–27).

In Iraq, breast cancer is the most frequently diagnosed malignancy and the first leading cause of cancer death among women where it accounts for one-third of registered cancers (28). The role of *CYP1A1m1* (T6235C) and *CYP1A1m2* (A4889G) as a risk in smoking women for breast cancer has not been examined in the local population. This work analyzed the genotype distribution and allele frequencies of *CYP1A1m1* and *CYP1A1m2* variants in Iraqi women with breast cancer. The study also investigated whether the active tobacco smoking status and duration alter the relationship between genotype distribution and the risk of breast cancer in Iraq.

## Subjects and Methods

### Study Subjects

This study consisted of 199 women with breast cancer and 160 cancer-free female controls. The histologically confirmed cases of breast cancer were recruited for approximately 2 years, 1st January 2015–30th January 2017 from Hospitals in Baghdad. The information on the demographic characteristics including medical and reporoductive histories of tobacco smoking for all participants about duration of their current and previous smoking habits. All procedures were conducted in accordance with the ethical standards of the Human Ethics Committee approval provided by the Ministry of Health (No. 6426). A written informed consent was obtained from all study participants after receiving approval of the experimental protocol by the ethics committee according to the Helsinki Declaration.

### Search Strategy and Bioinformatics Analyses of *CYP1A1* Polymorphisms

Thorough research was performed using NCBI-Resources, UCSC genome browser, and Ensemble databases. Search terms for the *CYP1A1* T6235C (*m1*) and *CYP1A1* A4889G (*m2*) polymorphisms and breast cancer risk were combined and included: tobacco smoke, polycyclic aromatic hydrocarbons’ cytochrome P4501A1 (*CYP1A1*: T6235C, rs4646903; A4889G, rs1048943), and breast cancer. Research papers, case studies, and review articles of relevant literature were manually searched to identify additional appropriate analyses.

The genomic criteria of *CYP1A1m1* and *m2* gene polymorphisms were searched in Ensemble, UCSC genome browsers, and GenBank databases. Several tools in the databases were used to retrieve and analyze the sequences, identify the strand (forward and/or reverse), flip the strand, and search for a particular sequence. The precise genomic map locations of the identified sequences were verified and updated to hg38 version of human genome sequence assembly by using the Gene Sorter and Table Browser tools of UCSC Genome Bioinformatics database.

### Nucleic Acid Isolation and *CYP1A1* Genotyping

Genomic DNA was extracted from whole blood samples of patients and controls using the QIAamp DNA mini Kit (Qiagen, USA) according to the manufacturer’s instructions. *CYP1A1* genotypes at the *m1* and *m2* sites were analyzed by PCR-based restriction fragment length polymorphism as described earlier with slight modification (11, 29, 30). The primers used for *m1* sites were M1F 5′-CAGTGAAGAGGTGTAGCCGCT-3′ and M1R 5′-TAGGAGTCTTGTCTCATGCCT 3′ while the primers used for *m2* site were M2F 5′-GGCTATCCTGCTGCAACGGGTGGAA-3′ and M2 R5′-CTGTCTCCCTCTGGTTACAGGAAG-3′. The M1F and M1R primers generated a product of 340 bp, while M2F and M2R generated a 204 bp product. Each PCR reaction mixture (25 µl) contained 100 ng template DNA, 0.2 µM each primer, 0.2 mM each dNTP, and 1.0 U Taq polymerase (Fermentas, Canada). To amplify the fragments containing *m1* and *m2* regions, the reaction involved an initial denaturation step of 7 min at 95°C, followed by 35 cycles of 30 s at 95°C, 1 min at 61°C for *m1* (63°C for *m2*) 35 s at 72°C and a final elongation step of 7 min at 72°C. The restriction enzyme *Msp1* (Fermentas, Canada) was used to distinguish the *m1* polymorphism as gain of an *Msp1* restriction site occurs in the polymorphic allele. The wild-type *m1* allele shows a single band representing the entire 340 bp fragment and variant allele results in two fragments of 200 and 140 bp. The restriction enzyme *BsrD1* (Fermentas, Canada) was used to distinguish the *m2* polymorphism from the same 204 bp product in two different reactions. The cleavage sites were lost in the case of the *m2* mutations and resulted in a single band, whereas the wild-type *m2* alleles generated 149 and 55 bp bands. The restriction-digested products were analyzed by electrophoresis on a 3% agarose gel containing ethidium bromide and visualized under a UV illuminator.

### Statistical Analyses

The Hardy–Weinberg (HW) equilibrium was tested using chi-squared (χ^2^) statistics for the goodness-of-fit (1 degree of freedom) between cases and controls. A multivariate analysis using the logistic regression was used to obtain the adjusted crude odds ratio (OR) with a 95% confidence interval (CI) and to assess the associations between variables of the *CYP1A1 m1* and *m2* genotypes between breast cancer cases, control group and smoking status. Covariates include age, smoking duration, and postmenopausal and familial history. For all statistical tests, the level of significance was two-sided at *p* < 0.05. All of the statistical analyses were performed with Statistical Analysis System software V.8 (SAS Institute, Cary, NC, USA).

## Results

### Genomic Context of *CYP1A1* Gene and the *m1* and *m2* Polymorphisms

**Figure 1:**
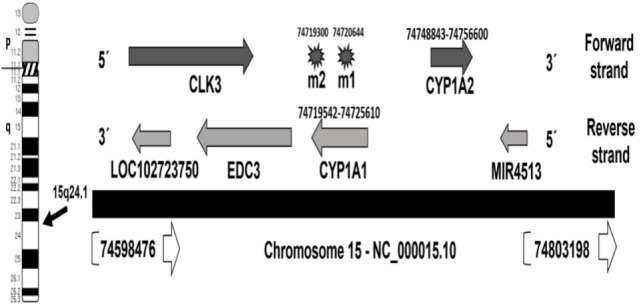
Map locations of *CYP1A1* locus and *m1* and *m2* polymorphisms on chromosome 15—NC_000015.10 region. The locations are updated to hg38 human genome assembly release (31).

The human *CYP1A1* locus is mapped in the negative strand of the long arm of chromosome 15 at 15q24.1, which occupies genomic space of 6,069 nucleotides at chr15: 74719542–74725610. This region is mapped in the chromosome 15-NC_000015.10 region that also contains *CYP1A2* on the forward strand (Figure 1). Careful analysis of the two polymorphisms of *CYP1A1, m1* and *m2*, in the databases showed that *m1* (also known A4889G) has SNP ID: rs1048943 with five synonyms, rs52810784, rs17861092, rs3188998, rs386513458, and VAR_001243. Whereas, *m2*, also known T6235C has SNP ID: rs4646903 of three synonyms, rs116877783, rs5030838, and rs17861083. The wild allele of *m1* codes isoleucine (I) or ancestral phenylalanine (F), while mutant *m1* allele leads to missense mutation resulting in an isoleucine (I) change to valine (V). Allele *m1* is mapped at chr15:74720644, which has four alleles (T/A/C/G). Genotype *m2* is the downstream gene variant which is mapped at chr15:74719300, with three alleles (A/G/T).

### Demographic Characteristics of the Study Subjects

This study included 199 women with breast cancer and 160 cancer-free controls. Table 1 shows the demographic and clinical characteristics of those subjects. There was no significant differences (*p* = 328) noted among patients (48.6 ± 2.0 years) and the healthy control group (47.8 ± 2.0 years) on the age risk factor. Whereas only the mean duration of smoking showed a significant difference (*t*-test, *p* = 0.029) between smoking women with breast cancer (30.1 ± 10.2 years) and the control group (21.4 ± 9.9 years). This may indicate that a longer smoking period may contribute to susceptible of women to breast carcinogenesis. All cancer patients were confirmed to have invasive ductal or lobular carcinoma by histopathology testing. The percentage of postmenopausal breast cancer patients was 46.7% which is very similar to the postmenopausal percentage of controls, 44.4%. This excludes the effect of estrogen exposure in our patient population as a significant factor.

**Table 1:** Demographic characteristics of women with breast cancer (patients) and healthy controls.

Parameter	Patients (%) (*n* = 199)	Controls (%) (*n* = 160)	*p*-Value
**Age (years)**			
≤40	44 (22.1)	42 (26.3)	0.396 NS
41–45	65 (32.7)	67 (41.9)	0.0416*
46–50	90 (45.2)	51 (31.9)	0.0317*
**Smoking status**			
Non-smoker	91 (45.7)	82 (51.3)	0.081 NS
**Smoker**			
Current	88 (44.2)	63 (39.4)	0.095 NS
Former	20 (10.1)	15 (9.4)	0.866 NS
Mean years smoked	(30.1 ± 10.2)	(21.4 ± 9.9)	(0.029*)
**Histological type**			
Invasive ductal carcinoma	91 (45.7)	NA	–
Invasive lobular carcinoma	108 (54.3)	NA	–
**Postmenopausal status**	93 (46.7)	71 (44.4)	0.612 NS
**Family history**			
First-degree relative with breast cancer	62 (31.2)	44 (27.5)	0.080 NS

*p < 0.05; NS, non-significant; n, number of patients = 199 and number of controls = 160; NA, not applicable.

### Genotyping of *CYP1A1m1* and *CYP1A1m2* Polymorphisms in Control and Breast Cancer Individuals

The results of genotype distribution of *CYP1A1m1T/C* and *CYP1A1m2A/G* displayed three genotypes with variable ratios in women with breast cancer and in the control group (Table 2). These results showed that 54.3% of women with breast cancer were carriers of CC mutant genotypes of *CYP1A1m1*, which was significantly higher than that observed in the control group (18.8%) (OR = 1.405, 95% CI = 0.89–1.63, *p* = 0.0083). Similarly, a highly significant difference was observed with the GG genotype of *CYP1A1m2* being prevalent in 48.7% of breast cancer patients and only 13.1% of the control group (OR = 1.379, 95% CI = 0.88–1.67, *p* = 0.0002). The heterozygous mutant genotypes for both *m1* and *m2* genes had significant differences between patients and controls (OR = 0.688, 95% CI = 0.87–160, *p* = 0.0431 and OR = 0.652, 95% CI = 0.92–163, *p* = 0.0259), respectively. On the other hand, the homozygous wild genotypes for *m1* (TT) and *m2* (AA) were significantly higher in the control groups (OR = 1.176, 95% CI = 0.92–1.62, *p* = 0.0027 and OR = 1.085, 95% CI: 0.92–1.92, *p* = 0.0097, respectively). This may reflect the protective role of the T (61%; OR = 0.28, 95% CI = 0.21–0.39, *p* = 0.0002) and A (67%; OR = 0.40, 95% CI = 0.29–0.55, *p* = 0.0002) alleles in the normal population against susceptibility to breast cancer. The C and G allele frequencies were higher (69 and 64%, OR = 3.53,95% CI = 2.59–4.81, *p* = 0.0002 and OR = 3.59, 95% CI = 2.0.75–5.40, *p* = 0.0001, respectively) in breast cancer patients (Table 2).

**Table 2:** Association between the *CYP1A1m1* and *CYP1A1m2* genotype and allele frequencies and breast cancer risk among patients and in the healthy controls.

Genotype	Patients (%)	Controls (%)	χ^2^ (*p*-value)	OR (95% CI)
***CYP1A1m1* (*T6235C*)**				
*w1/w1*^a^ (TT)	32 (16.1%)	66 (41.3%)	8.521** (0.0027)	1.176 (0.92–1.62)
*w1/m1*^a^ (TC)	59 (29.7%)	64 (40.0%)	4.277* (0.0431)	0.688 (0.87–1.60)
*m1/m1*^b^ (CC)	108 (54.3%)	30 (18.8%)	9.075** (0.0083)	1.405 (0.89–1.63)
**Alleles frequency**				
T	31%	61%	10.15**(0.0002)	0.28 (0.21–0.39)
C	69%	39%	11.16** (0.0002)	3.53 (2.59–4.81)
***CYP1A1m2* (A4889G)**				
*w2/w2*^a^ (AA)	40 (20.1%)	74 (46.3%)	7.933** (0.0097)	1.085 (0.92–1.92)
*w2/m2*^a^ (AG)	62 (31.2%)	65 (40.6%)	5.281* (0.0259)	0.652 (0.92–1.63)
*m2/m2*^b^ (GG)	97 (48.7%)	21 (13.1%)	10.26** (0.0002)	1.379 (0.88–1.67)
**Alleles frequency**				
A	36%	67%	11.47 (0.0002)	0.40 (0.29–0.55)
G	64%	33%	13.68** (0.0001)	3.59 (2.75–5.40)

*w^a^ refers to a wild-type genotype; m^b^ refers to a mutant genotype*.

*χ^2^ test for comparison between patients and controls*.

*p ≤ 0.05.

**p ≤ 0.01.

*OR, odds ratio; 95% CI, 95% confidence interval*.

*Total number of patients = 199 and the total number of controls = 160*.

### Prevalence of *CYP1A1* Polymorphisms in Smoker Patients and Healthy Controls

The influence of tobacco smoking on the distribution of *CYP1A1m1* and *m2* genotypes in both groups—patients and controls—revealed that *CYP1A1m1/m1* mutant CC genotype was significantly elevated in smoking women with breast cancer (75.9%) compared with the control group (10.3%) (OR = 1.607, 95% CI = 0.91–1.64, *p* = 0.0001) (Table 3). Similarly, the *CYP1A1m2/m2* mutant GG genotype was significantly higher in smoking women (83.4%) than the control group (7.7%) (OR = 1.841, 95% CI = 0.88–1.94, *p* = 0.0001). On the other hand, results of both *m1* and *m2* homozygous wild TT and AA genotypes were higher in control group women who smoked, 75.6 and 78.2% compared with women with breast cancer (4.6 and 3.7%) (OR = 1.659, 95% CI = 0.88–1.64, *p* = 0.0001 and OR = 1.702, 95% CI = 0.89–1.83, *p* = 0.0001, respectively). Similar patterns of C and G allele frequencies were also obtained among smoking patients (86 and 90%) [OR = 8.25 (3.27–11.44), *p* = 0.0002 and OR = 9.91 (4.35–16.41), respectively] (Table 3).

**Table 3:** Association of tobacco smoking on the *CYP1A1m1* and *CYP1A1m2* genotype and allele frequencies among women with breast cancer (patients) and healthy controls.

Genotype	Smoking groups		χ^2^ (*p*-value)	OR (95% CI)
	Patients (%) (*n* = 108)	Controls (%) (*n* = 78)		
***CYP1A1m1* (T6235C)**				
*w1/w1*^a^ (TT)	5 (4.6%)	59 (75.6%)	12.866** (0.0001)	1.659 (0.88–1.64)
*w1/m1*^a^ (TC)	21 (19.4%)	11 (14.1%)	1.935 NS (0.098)	0.173 (0.86–1.62)
*m1/m1*^b^ (CC)	82 (75.9%)	8 (10.3%)	12.257** (0.0001)	1.607 (0.91–1.64)
Alleles frequency				
T	14%	83%	17.5** (0.0002)	0.40 (0.21–0.62)
C	86%	17%	17.5** (0.0001)	8.25 (3.27–11,44)
***CYP1A1m2* (A4889G)**				
*w2/w2*^a^ (AA)	4 (3.7%)	61 (78.2%)	12.968** (0.0001)	1.702 (0.89–1.83)
*w2/m2*^a^ (AG)	14 (13.0%)	11 (14.1%)	1.02 NS (0.1755)	0.033 (0.90–1.61)
*m2/m2*^b^ (GG)	90 (83.3%)	6 (7.7%)	13.416** (0.0001)	1.841 (0.88–1.94)
Alleles frequency				
A	20%	85%	21.0** (0.0001)	0.2 (0.11–1.43)
G	80%	15%	21.0** (0.0001)	9.91 (4.35–16.41)

*w^a^ refers to a wild-type genotype; m^b^ refers to a mutant genotype*.

**p < 0.01.

*NS, non-significant; OR, odds ratio; 95% CI, 95% confidence interval; *n*, number of smoking patients = 108 (number includes current and former smokers), number of non-smoking patients = 91, and number of smoking controls = 78*.

### Interaction Between Tobacco Smoking and the *CYP1A1* Polymorphisms in Patients

The risk of breast cancer related *CYP1A1 m1* and *m2* polymorphisms were ascertained between smoking and non-smoking patients (Table 4). The results showed that *CYP1A1m1/m1* CC mutant genotype in smokers resulted in a greater risk for breast cancer (75.9%), than in non-smoking patients (21.9%) (OR: 1.802, 95% CI: 0.91–1.94, *p* = 0.0001). By contrast, the non-smoking patients carrying the homozygous wild TT genotype were more frequent (34%) than the smoking breast cancer patients (4.6%) (OR = 1.483, 95% CI = 0.89–1.61, *p* = 0.0001). Similarly, the *CYP1A1m2* genotype distribution showed that mutant GG genotype in smoking patients was at a higher risk for breast cancer (83.3%) in comparison with the same genotype in non-smoking patients (18.7%) (OR = 1.893; 95% CI = 0.90–1.96, *p* = 0.0001). While the homozygous wild-type AA genotype was more prevalent in non-smoking breast cancer patients (38.5%) than those in the smoking breast cancer patients (3.7%) (OR = 1.577, 95% CI = 0.87–1.63, *p* = 0.0001).

**Table 4:** Association of smoking risk with *CYP1A1 m1* and *CYP1A1m2* genotype distribution among women with breast cancer (patients only).

Genotype	Smokers (%) (*n* = 108)	Non-smokers (%) (*n* = 91)	*p*-Value	OR (95% CI)
***CYP1A1m1* genotype**				
*w1/w1*^a^ (TT)	5 (4.6%)	31 (34.0%)	0.0001**	1.483 (0.89–1.61)
*m1/m1*^b^ (CC)	82 (75.9%)	20 (21.9%)	0.0001**	1.802 (0.91–1.94)
***CYP1A1m2* genotype**				
*w2/w2*^a^ (AA)	4 (3.7%)	35 (38.5%)	0.0001**	1.577 (0.87–1.63)
*m2/m2*^b^ (GG)	90 (83.3%)	17 (18.7%)	0.0001**	1.893 (0.90–1.96)

*w^a^ refers to a wild-type genotype; m^b^ refers to a mutant genotype*.

*OR, odds ratio; 95% CI, 95% confidence interval for comparison between smokers and non-smoker patients*.

**p < 0.01.

*Total number of smoking (*n* = 108, number includes current and former smokers) and non-smoker patients (*n* = 91)*.

## Discussion

In this case–control study, we investigated the correlation between tobacco smoking and gene polymorphisms of *CYP1A1m1* T6345C and *CYP1A1m2* A4889G with breast cancer risk in women from Iraq. The results demonstrated that gene polymorphisms of both *CYP1A1m1* CC and *CYP1A1m2* GG mutant genotypes and C, G allele frequencies were significantly associated with a higher risk for breast cancer when compared with healthy controls. The patient and control groups were in HW equilibrium at both *CYP1A1m1* and *m2*. In addition, the interaction between these two polymorphic mutant genotypes and tobacco smoking was strongly correlated with a higher risk of breast cancer among tobacco smoking women in Iraq. This interaction may highlight the significant influence of the *CYP1A1* gene polymorphisms on various risk factors, such as tobacco smoking, associated with breast cancer. In general, the *CYP1A1* polymorphisms may vary between populations due to differences in race or ethnicity of other populations (32). The polymorphism was evident in many different race and ethnic groups as in African-American (10, 12, 15), Chinese (14, 16), Siberians (17), Canadians (19), Kashmiri and northern Indians (30, 33), Hispanic-non-Hispanic white women (32), Egyptian Arabs [Ref. (34) and this study], and Japanese (35). On the other hand, the TT and AA wild homozygous genotypes as well as T and A allele frequencies were significantly higher among the control group which became more prevalent among smoking women with breast cancer. Whether these mutation were protective or harmful, the *m2* variant caused a conservative missense and neutral substitution from isoleucine to valine (both aliphatic) at position 426 in the mutant protein which assessed as non-detrimental variant assessed by online service PolyPhen 2 (http://genetics.bwh.harvard.edu/pph2), whereas the *m1* variant is considered as a polymorphism variant with a T>C substitution at 6235 position in the 3′ non-coding region.

The role of *CYP1A1* polymorphisms in breast cancer risk in individuals, however, has been conflicting. Some studies have shown that *CYP1A1* polymorphisms are significantly associated with breast cancer risk (13, 34, 36) consistent with this study. In this study, patients with either G or C allele carriers were approximately equally susceptible to breast cancer and the G and C allele carriers were under represented in the control group. These distinguished observations became more obvious among smoking women with breast cancer. Others have reported that the increased risk of breast cancer was only among G mutant allele, but not C allele carriers (37), and influenced by smoking status (23, 33, 38). By contrast, others have failed to show such an association (15). Conversely, *CYP1A1* gene polymorphisms were associated with a lower risk of breast cancer among Japanese women (35). Moreover, there is no clear association between the *CYP1A1* gene polymorphism and breast cancer risk in various parts of the world (15, 16). These inconsistent results may be attributed to different sample sizes and ethnic variations among studied populations, as well as exposure to various environmental risk factors. The mechanism of *CYP1A1* gene polymorphism effect on cancer induction can be attributed to different pathways such as phase 1 bioactivation of xenobiotics and involvement in the metabolisms of estrogen (converting the metabolites into carcinogens) that leads to elevating the risk of breast cancer (17), lung cancer (29, 30), and renal cell cancer (39), or due to the activation of mammary carcinogens by tobacco metabolites (40).

The increased risk of breast cancer among long duration smoking patients in Iraq was associated with both *CYP1A1* C and G allele carriers and could also be attributed to recurrent war episodes and the socioeconomic consequences, for example, continuous fear of child abduction during the recent civil unrest, putting additional burden and stress on women in Iraq. In addition, exposure to seasonal dust-storms, inhalation of chemicals, pollution of air by electric generators across urban suburbs and carcinogens which may increase the level of *CYP1A1* expression/mutation in the target tissues. Activation *via* the aryl hydrocarbon receptor (AhR), by affecting cell signaling pathways, might reflect the role of the AhR in tumor progression (41, 42). The mechanism of interaction between AhR and *CYP1A1* or CYP2, where both are involved in the metabolism of estrogen which may lead to alteration in steroid levels modulating bioactivation of therapeutic agents and xenobiotics and elevating the risk of breast cancer in smoking women. A larger sample size study including smoking women living in rural areas women is needed to unravel the complex interplay effects between gene polymorphisms and tobacco smoking and to compare between local environmental factors.

## Conclusion

Results of this study showed a statistically significant association between the polymorphisms of *CYP1A1m1* CC and *m2* GG genotypes and C and G alleles with the increased risk of women of breast cancer in Iraq. Moreover, this strong association at both genotype and allele levels was exacerbated among tobacco smoking women with breast cancer. On the other hand, the wild TT, AA genotypes, and T and A allele carriers were significantly prevalent among healthy non-cancerous women which persist among smoking women as well. Therefore, T and A alleles may play a protective role against breast cancer in this population. The predominance of C and G alleles among tobacco smoking women with breast cancer may suggest a role in breast cancer susceptibility among Iraqi women which could have prognostic implications in breast cancer.

## Ethics Statement

Ethical clearance was obtained from the Ministry of Health of Iraq Government. The study participants were informed about the study in Arabic, including the purpose of the study. Only those who agreed and signed the informed consent were included. PI ensured partcipants’ confidentiality and anonumity as well as no risk or harm involved.

## Author Contributions

All authors listed have made a substantial, direct, and intellectual contribution to the work and approved it for publication.

## Conflict of Interest Statement

The authors declare that the research was conducted in the absence of any commercial or financial relationships that could be construed as a potential conflict of interest.
